# *In vitro* potentiation of doxorubicin by unseeded controlled non-inertial ultrasound cavitation

**DOI:** 10.1038/s41598-019-51785-7

**Published:** 2019-10-30

**Authors:** Cécile Fant, Maxime Lafond, Bernadette Rogez, Ivan Suarez Castellanos, Jacqueline Ngo, Jean-Louis Mestas, Frédéric Padilla, Cyril Lafon

**Affiliations:** 1 LabTAU, INSERM, Centre Léon Bérard, Université Lyon 1, Univ-Lyon, F-69003 Lyon, France; 2 Present Address: Department of Internal Medicine, University of Cincinnati, 2600 Clifton Ave, Cincinnati, OH 45220 USA; 3 University of Lille, building SN3, INSERM U908 “Cell plasticity and Cancer”, 59655 Villeneuve d’Ascq, France; 4 OCR (Oncovet Clinical Research), Parc Eurasanté, Lille Métropole, 80 rue Docteur Yersin, 59120 Loos, France; 5 Department of Radiation Oncology, University of Virginia School of Medicine, Charlottesville, VA USA; 6 Focused Ultrasound Foundation, 1230 Cedars Court, Suite 206, Charlottesville, VA USA

**Keywords:** Drug delivery, Biomedical engineering

## Abstract

Ultrasound-generated non-inertial cavitation has the ability to potentiate the therapeutic effects of cytotoxic drugs. We report a novel strategy to induce and regulate unseeded (without nucleation agents) non-inertial cavitation, where cavitation is initiated, monitored and regulated using a confocal ultrasound setup controlled by an instrumentation platform and a PC programmed feedback control loop. We demonstrate, using 4T1 murine mammary carcinoma as model cell line, that unseeded non-inertial cavitation potentiates the cytotoxicity of doxorubicin, one of the most potent drugs used in the treatment of solid tumors including breast cancer. Combined treatment with doxorubicin and unseeded non-inertial cavitation significantly reduced cell viability and proliferation at 72 h. A mechanistic study of the potential mechanisms of action of the combined treatment identified the presence of cavitation as required to enhance doxorubicin efficacy, but ruled out the influence of changes in doxorubicin uptake, temperature increase, hydroxyl radical production and nuclear membrane modifications on the treatment outcome. The developed strategy for the reproducible generation and maintenance of unseeded cavitation makes it an attractive method as potential preclinical and clinical treatment modality to locally potentiate doxorubicin.

## Introduction

Doxorubicin (DXR), also termed Adriamycin, is a chemotherapeutic agent widely used against many malignancies including solid tumors (breast, endometrium, ovary, thyroid, lung, bladder, stomach and sarcomas of the bone) and hematological malignancies (lymphoma, acute lymphoblastic and myeloblastic leukemia)^1,2^. Despite its widespread use, DXR is associated with acute or chronic toxicity in healthy tissues, such as cumulative cardiotoxicity, hematotoxicity or secondary risk of acute myeloid leukaemia^3^. The development of resistances in tumor cells, linked to P-glycoprotein and topoisomerase II, can hamper the DXR therapeutic efficacy^4^. To overcome these limitations, developing an efficient strategy to potentiate the action of DXR is required to improve the efficacy of chemotherapy while minimizing the therapeutic dose administered to patients^5^.

Ultrasound (US) can interact with chemotherapeutic agents to maximize their biological actions and enhance tumor cell death, while causing minimal damage to surrounding healthy tissues^6^. More specifically, the synergetic therapeutic effect of US and DXR on tumor cells was observed in several studies^7–9^. Under proper settings, US can create gas bodies in tissues that will act as oscillating nuclei that shrink in response to positive pressures and expand under negative pressures, a phenomenon called cavitation. Cavitation is usually described in two forms: non-inertial cavitation describes a cyclic, sustainable and nonlinear expansion and contraction of a gas bubble, whereas inertial cavitation refers to the violent collapse of bubbles^10^. Non-inertial cavitation is oftentimes termed as stable cavitation, which should be avoided in work related to cavitation control to prevent confusion with situations where stable inertial cavitation, or transient non-inertial cavitation can be obtained. For therapeutic applications of US, cavitation activity is usually enhanced by adding cavitation nuclei such as ultrasound contrast agents (UCA), gas bubbles of 1–4 µm in diameter stabilized by a shell, typically composed by phospholipids, that remain in the blood stream^11,12^ with intended mechanical effects resulting in bioeffects such as sonoporation or enhancement of vascular permeability. The later mechanism is the subject of current preclinical and clinical studies aiming at transiently opening the blood brain barrier (BBB) for drug delivery in a safe and reproducible way^13–16^. Echocontrast agents on the gaseous form are limited to the micrometric scale to remain thermodynamically stable^17^ and thus do not permit to nucleate cavitation in areas remote from the tumor vasculature. Alternative for such agents in the sub-micron scale as nanobubbles or nanocups are being investigated^18–21^.

Cavitation is one of the ultrasound-associated mechanism that creates numerous biological effects and might affect drug activity^22,23^. Non-inertial cavitation can permeabilize transiently the plasma membrane by creating resealable nano-sized pores, allowing transportation of molecules across the membrane^24–26^, a process called sonoporation^27^. Cavitation can cause intracellular effects and localize macromolecules directly into the nucleus^28,29^, through the augmentation of nuclear pores and a change in their distribution throughout the nuclear membrane, which could potentiate the action of intercalant DNA agents such as DXR. US can affect DNA structure as well as its function by inducing single- and double strand breaks (DSBs)^30^. These alterations could lead to enhanced cell apoptosis in the presence of DXR, as well as changes in cell morphology (such as membrane poration or organelle destruction) and in cell nuclei^31,32^. Ultrasound interaction with cells and tissue can also lead to US-induced mild hyperthermia (37 °C to 41 °C), which can also increase drug penetration in tumor cell suspensions^33^. US cavitation can generate free radicals, being another potential mechanism for an enhanced DXR cytotoxicity^34,35^.

We hypothesize that combining DXR with unseeded (without nucleation agents) non-inertial cavitation would enhance its therapeutic efficacy. As cavitation induced without the need of UCA can potentially be generated in all tissues, and not only the vasculature space reached by UCA, this method can provide a higher versatility compared to UCA-based cavitation. Moreover, developing a method to generate unseeded cavitation with high reproducibility over long period of time can overcome another hurdle associated with the use of UCA, which is the requirement to have UCA infusion in order to generate constant level of cavitation^36^. Alternating between high amplitudes US pulses to generate bubble cloud and low amplitude pulses to sustain its oscillation has been reported to show benefits on cavitation activity^37^. In our experimental setup, the method is used to generate and maintain cavitation activity, using monitoring of cavitation activity to dynamically dictate the alternation between the two power modes.

We report in this study the generation and maintenance of non-inertial cavitation *in vitro* with high reproducibility, and without the use of UCA, by using two US power modes adequately alternated by a feedback loop. This unseeded non-inertial cavitation can act synergistically with DXR to affect 4T1 cells viability and proliferation *in vitro*. We investigated the underlying mechanisms enhancing the therapeutic effect: changes in cell morphology such as membrane poration, organelle destruction or formation of nuclear pore complex; increased DXR internalization due to sonoporation; heating and free radicals formation.

## Results

### Reproducibility of non-inertial cavitation

**Figure 1:**
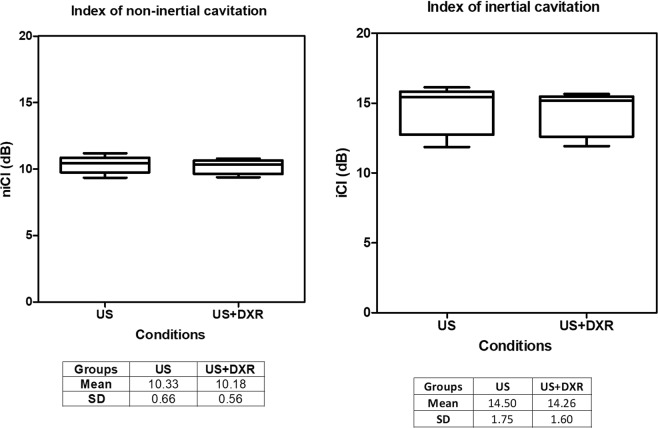
Reproducibility of non-inertial cavitation. niCI and iCI values obtained for US and US + DXR groups when each experiment was considered as an independent measure. Figure shows that non-inertial cavitation was generated in a controlled and reproducible manner.

To quantify the level of non-inertial and inertial cavitation, two cavitation indexes (CI) were defined based on specific acoustic features: the non-inertial cavitation index – niCI (dB), calculated as the difference between the maximum power level in the 540–560 kHz sub-harmonic range and the mean base level obtained in the 560–600 kHz range – and the inertial cavitation index – iCI (dB), calculated as the difference between the average of the entire frequency spectrum in dB in the 0.1–7.1 MHz range and its base level. On average over the different sets of experiments, the niCI was found well above the non-inertial cavitation threshold of 3 dB (Fig. 1). Similar niCI were obtained in the presence or absence of DXR (niCI of 10.32 ± 0.93 in the absence of DXR, 10.16 ± 0.63 in the presence of DXR). These values were also reproducible between experiments, confirming the generation and the control of non-inertial cavitation activity (Fig. 1). Non-inertial cavitation was generated in exposed samples with approximately 2.7% of bursting pulses to maintain cavitation all along the exposure duration. The iCI was 14.55 ± 1.69 in the absence of DXR, and 14.23 ± 1.54 in the presence of DXR. These values are below the iCI threshold for inertial cavitation initiation (Fig. 11). The inertial cavitation activity is residual from the high amplitude bursting pulses.

### *In vitro* enhanced cytotoxicity effect

#### Cell viability

**Figure 2:**
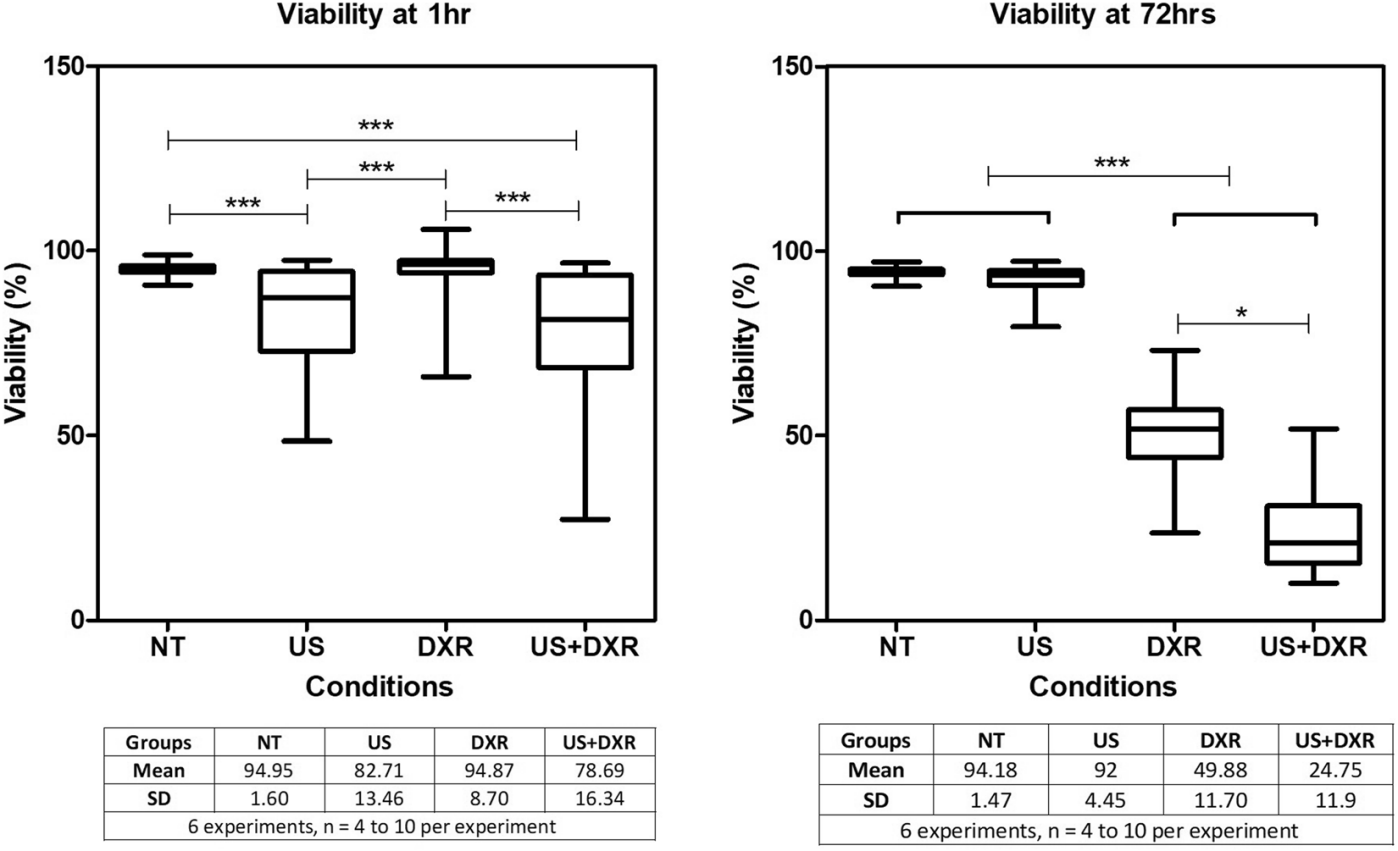
Cell viability at 1 h and 72 h of cells exposed to DXR alone, controlled non-inertial cavitation alone, or the combination of the two (n = 34 per group). This experiment demonstrates the synergy of the combined treatment on cell viability.

The cytotoxicity of the combined treatment with doxorubicin and non-inertial cavitation was assessed *in vitro* 1 h and 72 h after treatment. Sonication significantly decreased the cell viability at 1 h of approximately 15% (p_value_ < 0.0001, post-hoc test p-value indicated on Fig. 2). At 72 h, there was a statistically significant difference between the treatment groups (p_value_ < 0.0001, post-hoc test p-value indicated on Fig. 2). DXR decreased the cell viability compared to controls and US. These results are consistent with the mortality rate and kinetics expected after a treatment with doxorubicin at 400 ng/mL, as cell death caused by DXR is expected to occur only after 24 to 48 h^38^. In the US + DXR group, cell viability was significantly lower than in the DXR group at 72 h with a drop of cell viability of 50% compared to DXR alone.

#### Cell proliferation

**Figure 3:**
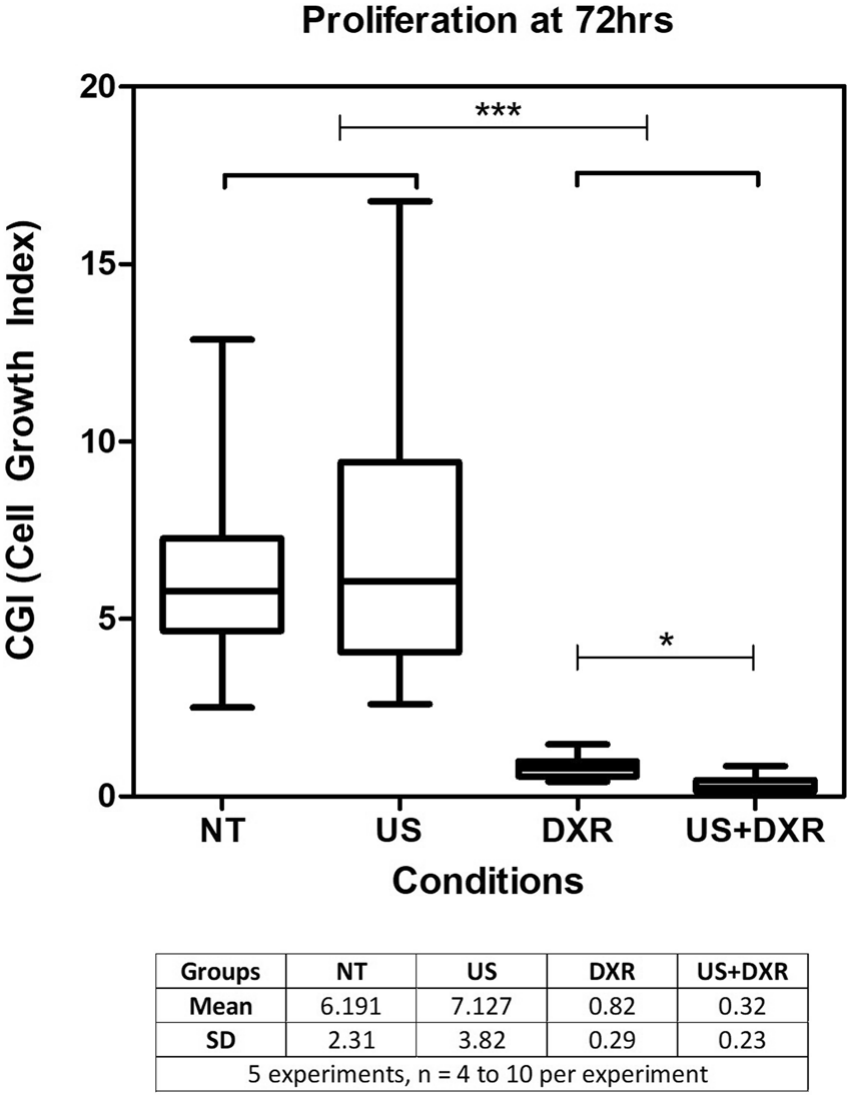
Effect of US, DXR and combined treatment on the 4T1 cell proliferation (n = 30 per group). Cells were counted at t = 0 and 72 h. This experiment demonstrates the synergy of the combined treatment on cell proliferation.

Cell proliferation was quantified over time with a cell growth index (CGI), the ratio of cell number at 72 h over cell number at the time of plating. At 72 h, the CGI was close to 6 for controls and US groups, likely limited by having reached confluence in the culture wells. In DXR treated samples, CGI was 0.82 ± 0.29 after 72 h. In US + DXR samples, the CGI was 0.32 ± 0.23 after 72 h. The CGI at 72 h was significantly lower in the US + DXR group compared to DXR alone (p_value_ < 0.0001, post-hoc test p-value indicated on Fig. 3).

### Mechanistic study

#### Effect of the presence of cavitation

Our system uses high pressure bursting pulses to generate cavitation, and then lower pressure pulses to maintain non-inertial cavitation activity. When the bursting pulses were not applied, no cavitation could be detected during the treatment as quantified by the very low average levels of the cavitation indexes (niCI = 1.81 ± 2.81, iCI = 9.87 ± 0.22). When boosting pulses were applied, the levels of cavitation indexes reached values associated with non-inertial cavitation activity (niCI = 8.07 ± 3.19; iCI = 12.06 ± 1.21).

**Figure 4:**
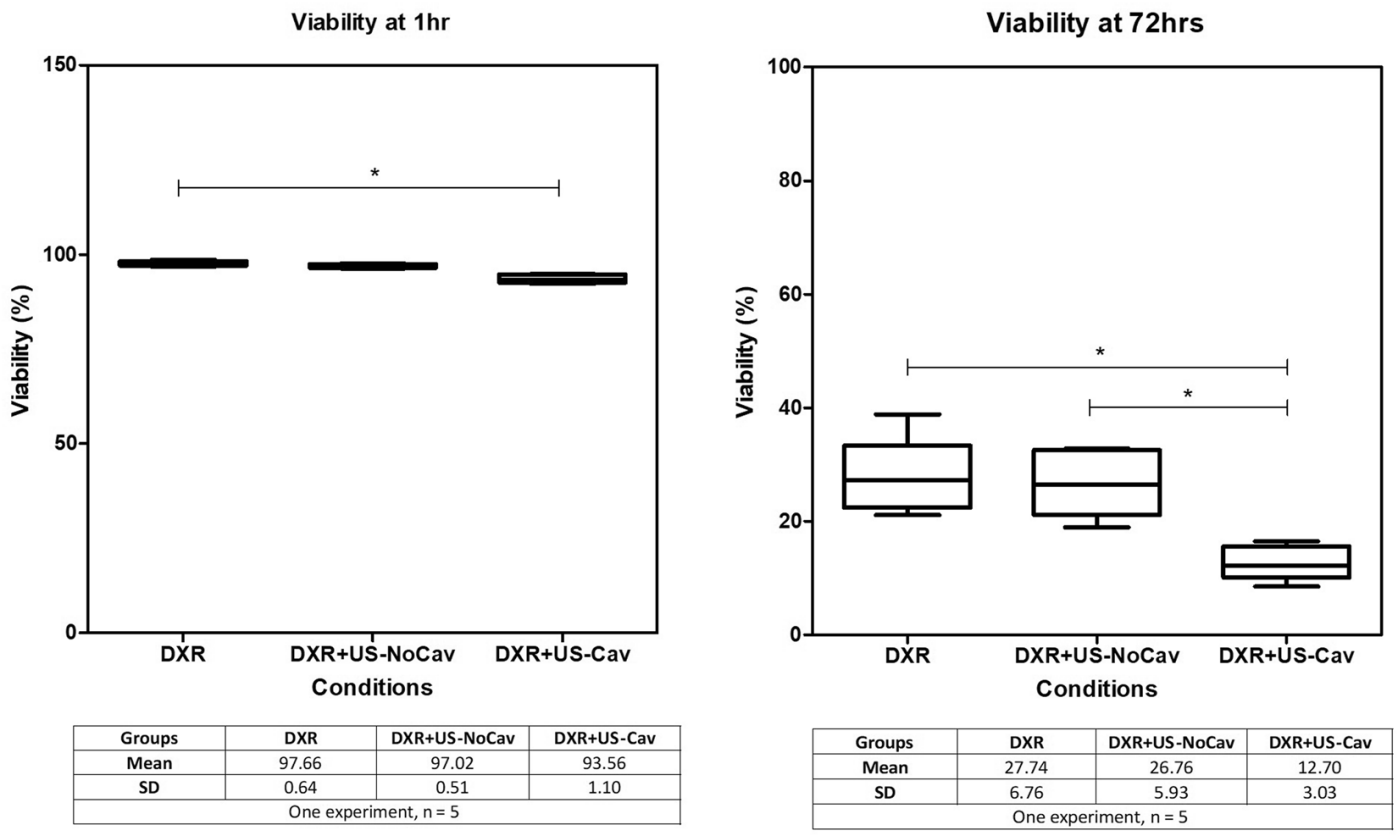
Effect of cavitation (n = 5 per group). Therapeutic efficacy (viability) at 1 h and 72 h of DXR alone (DXR) and in combination with ultrasound without cavitation (DXR + US-NoCav) or with controlled non-inertial cavitation (DXR + US-Cav). This experiment demonstrates the impact of non-inertial cavitation on cell viability.

In DXR groups, the cell viability with and without sonication was similar in the absence of cavitation at any time point (Fig. 4).

DXR + US-Cav treatment decreased the cell viability compared to DXR alone at 1 h (p_value_ = 0.0048, post-hoc test p-value indicated on Fig. 4) and to DXR alone or DXR + US-NoCav at 72 h (p_value_ = 0.009, post-hoc test p-value indicated on Fig. 4), demonstrating that cavitation is required for inducing the therapeutic effect observed.

#### DXR internalization

**Figure 5:**
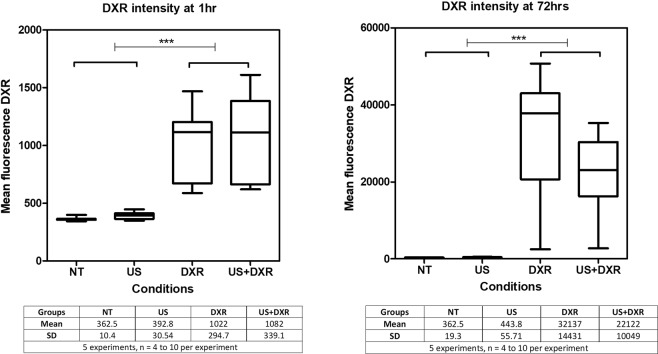
Measure of the mean intensity of DXR in DXR and US + DXR groups (n = 30 per group). Measures were done at 1 h and 72 h.

The fluorescence intensity of DXR was similar between DXR and US + DXR groups at the two time points, suggesting that the drug uptake was similar with and without unseeded cavitation (Fig. 5).

#### Effect of temperature

**Figure 6:**
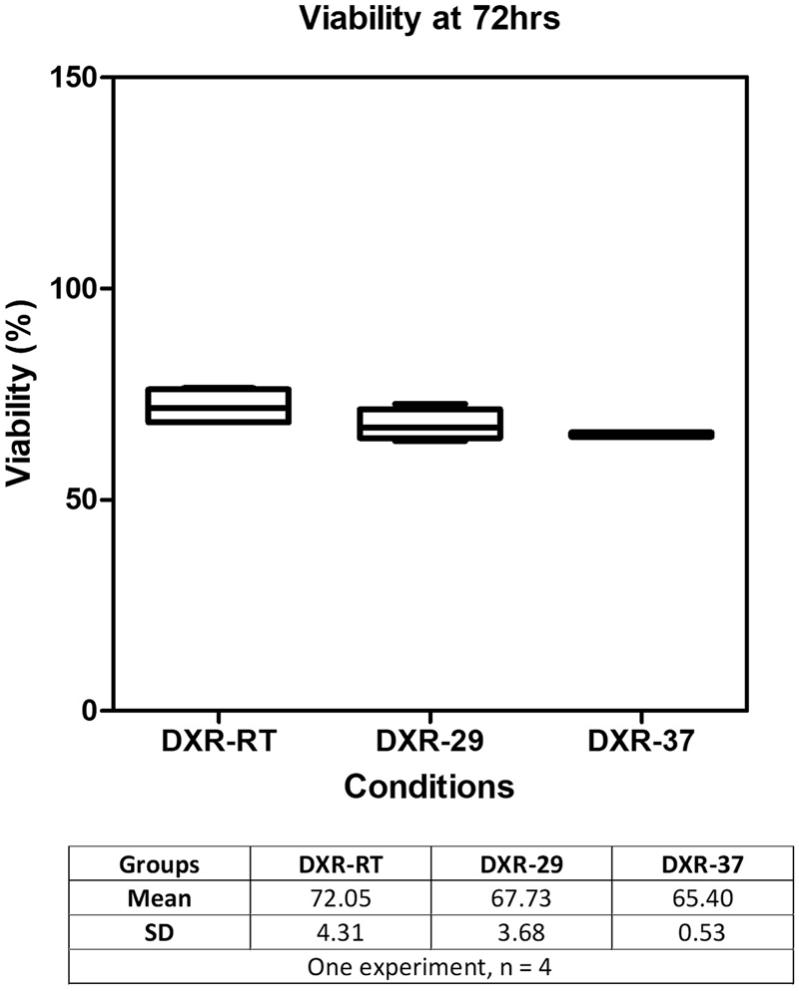
Effect of temperature (n = 4 per group). Cell viability 72 h after exposure to either DXR alone (DXR), water bath to mimic US heating (bath 29 °C – DXR-29) or stronger heating (bath 37 °C – DXR-37). There is no statistically significant difference in cell viability between the three conditions.

The US-induced hyperthermia was mimicked using a water bath heated at 29 °C or 37 °C. No significant difference in cell death after 72 h (p_value_ = 0.51) was observed between the heated and non-heated groups (Fig. 6). This suggests that the transient US-induced temperature elevation did not enhance the combined DXR efficacy in our study.

#### Effect of reactive oxygen species

**Figure 7:**
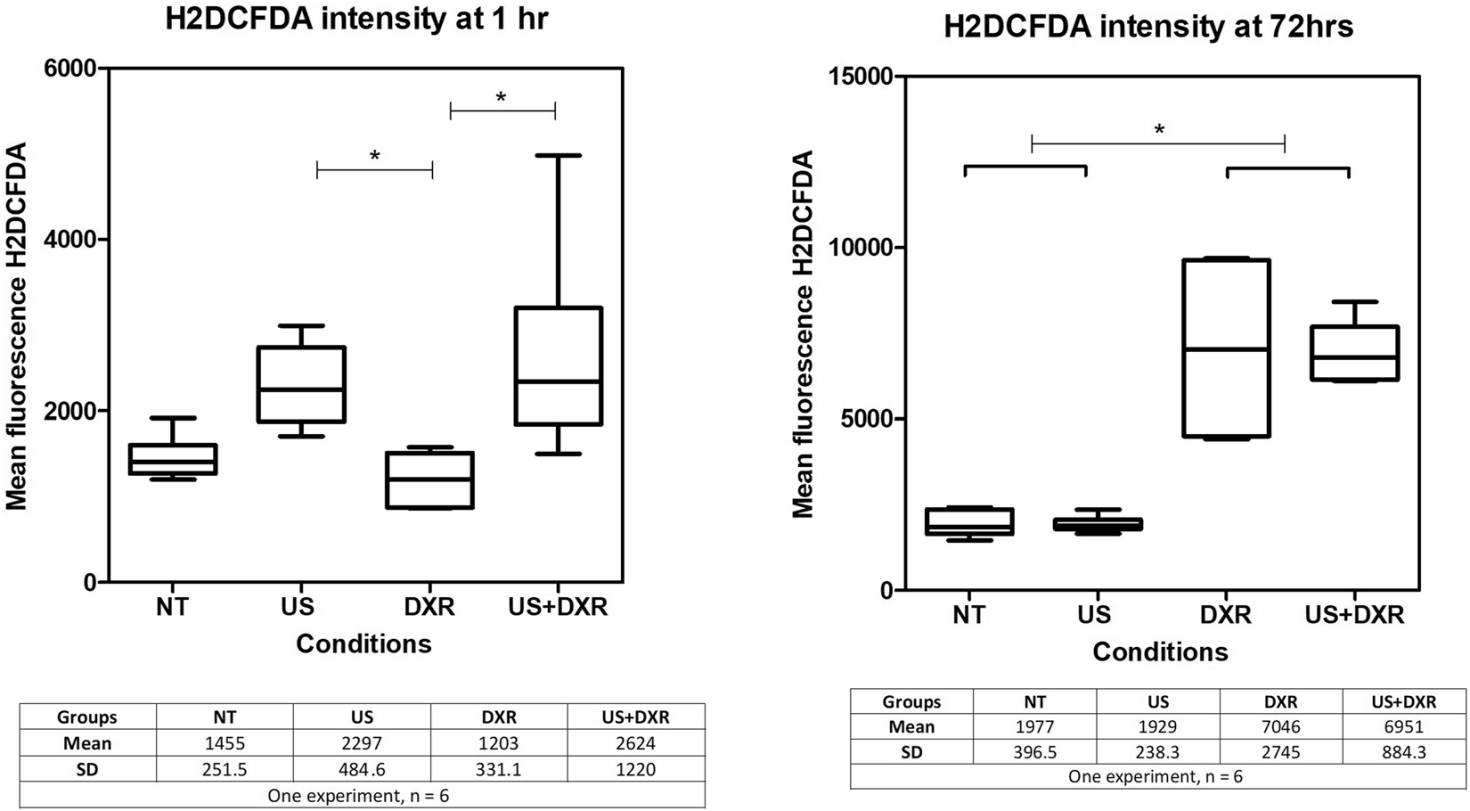
Mean intensity of fluorescence of H2DCFDA at 1 h and 72 h in the four groups (n = 6 per group). A significant difference is observed between sonicated groups and DXR group at 1 h, and between groups with or without DXR at 72 h.

The mean quantity of ROS in each sample was assessed using the specific ROS marker H2DCFDA. At 1 h, the mean quantity of ROS was significantly higher in sonicated groups than in DXR group and tend to be higher compared to non-treated group (p_value_ = 0.0015, post-hoc test p-value indicated on Fig. 7). At 72 h, the mean quantity of ROS was significantly larger for groups with DXR than for groups without DXR (p_value_ = 0.0009, post-hoc test p-value indicated on Fig. 7). The data may suggest two different mechanisms of ROS production, one short term due to acoustic cavitation, and one long term due to DXR. The results concerning mean intracellular quantity of doxorubicin, cell viability and growth index in each group were similar to those obtained in the previous experiments.

**Figure 8:**
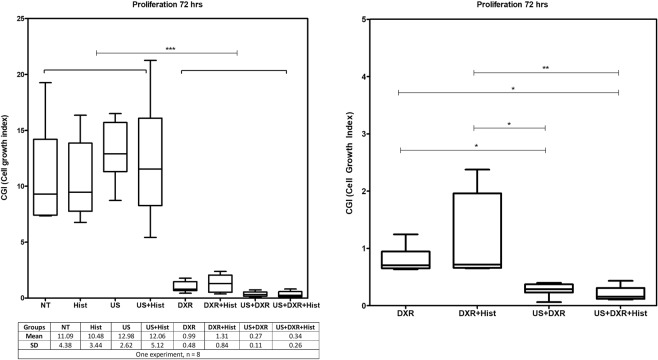
Effect of ROS on cell cytotoxicity (n = 8 per group). (**A**) Cell proliferation rate while adding Histidine to block the effect of the ROS. (**B**) Cell proliferation rate for DXR, DXR + Hist, US + DXR and US + DXR + Hist groups ROS scavenging did not affect cell proliferation in any of the conditions tested.

To assess whether ROS production contributes to cytotoxicity, 4T1 cells were cultured in the presence of a hydroxyl scavenger (L-Histidine), and cell viability assessed at 1 h and 72 h. For all the treatment conditions tested, the viability was not significantly affected by ROS scavenging and were similar to those obtained in the previous set of experiments. ROS scavenging did not affect cell proliferation at 72 h in any of the condition tested (p_value_ < 0.0001, post-hoc test p-value indicated on Fig. 8), nor did it affect changes in viability induced by ultrasound at 1 h, nor the increased cytotoxicity of the combined treatment US + DXR compared to DXR alone at 72 h.

#### Bioeffects of the US on cell membranes

**Figure 9:**
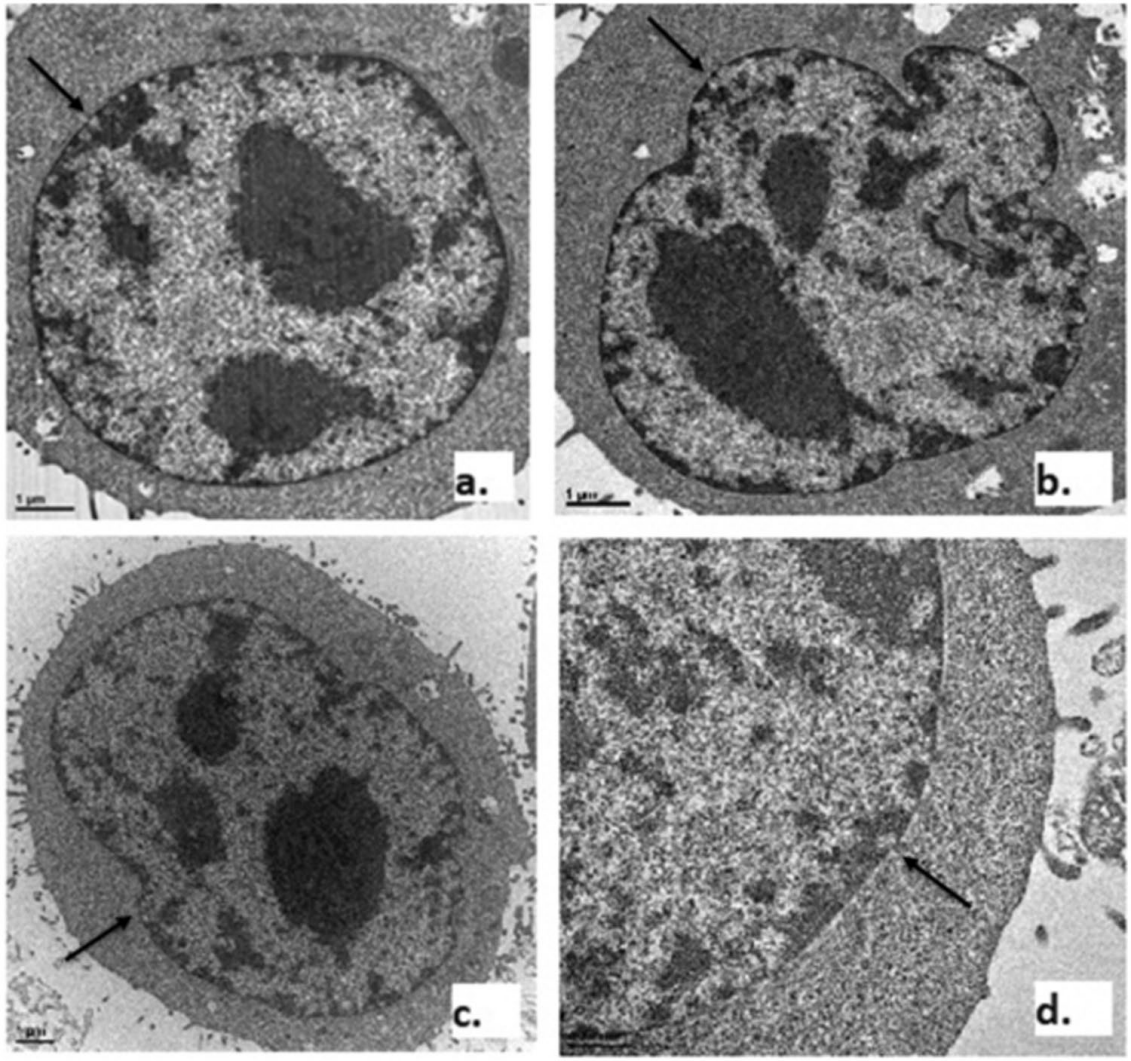
Effect of US on cell membranes (n = 4 per group). Images examples of pores in the cell nucleus in non-treated (x10) (**a**), doxorubicin (x15) (**b**), sonicated (x10) (**c**), and ultrasound + doxorubicin (x30) (**d**) cells. Pores are pointed by the black arrows.

Confocal microscopy observations of cells did not show any morphological differences between sonicated and non-sonicated cells, either at the levels of the entire cell, cell membrane, the nuclear membrane or the cell organelles. The quantity of nuclear pore complexes was similar in all samples (24 ± 2 per cell). Typical example images of these pores are shown on Fig. 9.

## Discussion

The aim of this study was to evaluate DXR potentiation by unseeded non-inertial cavitation, to induce cell death in murine 4T1 mammary tumor cells *in vitro*. Using a confocal device, we first designed a feedback loop that alternates conditionally between two exposure levels that either generate bubbles or maintain the non-inertial cavitation activity. The parameters were optimized so that reproducible non-inertial cavitation exposures could be applied. In a pilot study on 4T1 tumors in mice (unpublished data), we were also able to generate and maintain non-inertial cavitation in tumor area using a different set of parameters (DC 15%, PRF 100 HZ). This experiment provides encouraging leads for our regulation system to efficiently generate and maintain non-inertial cavitation *in vivo*.

We studied a potential synergetic effect of DXR and US. The action of US combined to that of DXR had both a higher and a faster therapeutic effect on tumor cells, therefore supporting a potential synergy. The experiments showed that ultrasound did not induce delayed cell mortality, as the immediate mortality assessed at 1 h did not have any influence on the results at 72 h. Furthermore, cells that did not die immediately after US treatment proliferated normally; cell growth in the US group was similar to that in the control group. No therapeutic effect could be observed in the absence of cavitation at 72 h, for the same amount of energy deposition in the exposed samples. At 1 h, the viability in the sonicated group with non-inertial cavitation was significantly lower than in the control group and lower than the sonicated group without non-inertial cavitation, although this last difference was not statistically significant probably due to low number of samples and low sensitivity of non-parametric test. This further highlights the key role of cavitation in mechanism of action and bioeffects of US.

To investigate the mechanisms by which cavitation can enhance cytotoxicity of DXR in this *in vitro* experimental settings, several factors were independently investigated. One of the most important and intriguing point is highlighted by the absence of increased DXR internalization after US treatment. Indeed, one of the initial rational for this work was that US-induced cavitation would enhance the intracellular delivery of drugs by transiently increasing the cell membrane permeability^39^. The increased permeability temporal window has been studied in 4T1 cells and estimated to be 3 h^40,41^. In the current experimental settings, there was no observable difference in DXR internalization between DXR and US + DXR group at short-term (1 h) or long term (72 h) post-treatment. One of the possible explanations for the absence of enhanced internalization in our specific experimental settings lies in the post-treatment flow cytometry protocol that happened after cells have been exposed for 1.5 to 2 h to DXR. Because doxorubicin transport across cell membranes takes place by simple Fickian diffusion^42^ and the equilibrium between intracellular and extracellular concentration of DXR is rapidly achieved, all cells, sonicated or not, may have reached intracellular peak concentration at the time of flow cytometry analysis.

The effect of temperature elevation caused in our *in vitro* experiments by US exposure was investigated. Although the kinetics of the temperature elevation caused by ultrasound exposure was not exactly reproduced inside a heated water bath, both US treated and warmed samples were allowed to reach similar maximum temperature of 29 °C. In a separate condition, samples were even heated by a 37 °C water bath for 30 s. None of the heating experiments resulted in any significant therapeutic effects of DXR compared to non-heated conditions, and temperature elevation was ruled out as a causative mechanism of the observed synergetic effect between US and DXR. It may be assumed that a 30 s temperature elevation of 2 °C as observed in our experiment is too short to significantly influence cell metabolism. Another possible explanation lies in our methodology as cells were always in presence of free DXR. Temperature may influence DXR activity and cytotoxicity, as mentioned in several studies^43,44^. However, in these studies, DXR was removed from the cell medium after exposure to investigate the impact of temperature on its kinetics. In our experiments, because cells were exposed to DXR after each treatment, the change in temperature did not impact cytotoxicity at 72 h. A recent clinical study reported increased intratumoral drug delivery after ultrasound-mediated hyperthermia and injection of lyso-thermosensitive liposomal DXR^45^. However, in our *in vitro* experimental setting, and as reported *in vivo* by Lyon *et al*., it is unlikely that addition of ultrasound-mediated hyperthermia to free doxorubicin would significantly increase cytotoxicity.

Cavitation triggers the generation of reactive oxygen species (ROS)-derived radicals, which can initiate a chain peroxidation of cell membrane lipids^46^. ROS generation is one of the DXR ways of action. Thus, the ROS produced by cavitation may add up and increase the overall cytotoxicity of the combined treatment. This strategy is known as sonodynamic therapy^47^. ROS production was quantified using H2DCFDA. Sonication tend to increase ROS formation at 1 h, mostly likely due to stressed cells after sonication. ROS quantities were increased for DXR-treated groups at 72 h. This suggests that DXR was the major regulator of ROS production in our treatment scheme^48^. However, their inhibition with histidine did not affect cell proliferation at 72 h in any of the condition tested, and the role of ROS production in our settings remains to be clarified.

Possible opening of nuclear pore complexes upon ultrasound exposure was also investigated. Such opening could lead to increased uptake of DXR or changes and/or alterations of the cell homeostasis, such as endoplasmic reticulum stress or different mitochondria potential membrane. These effects have been reported following ultrasound cavitation^23,49–52^, but no difference could be observed between sonicated and non-sonicated cells in our study. Despite the previous uncertainty on whether the cytometry analysis is biased or not by a potential DXR peak concentration reached in both DXR and US + DXR groups, these morphological observations reinforce the hypothesis of sonoporation not being a responsible mechanism of US-enhancement of DXR in our study.

Our mechanistic investigation did not provide a clear understanding of what mechanism is responsible for the observed synergy between DXR and US non-inertial cavitation. We propose that DXR-US treatment affecting DNA damage could be a possible mechanism, as ultrasound has been reported to be able to induce DNA double-strand breaks (DSBs)^28^, and DXR intercalation with the mitochondrial^53^ or the nuclear DNA double helix causes inhibition of topoisomerase II leading to DSBs^28,54^.

In conclusion, we designed an efficient strategy to produce controlled and reproducible non-inertial cavitation. We showed that unseeded non-inertial cavitation potentiates the cytotoxic effect of doxorubicin on cell viability and proliferation in 4T1 tumor cells *in vitro*. Cavitation was identified as necessary to enhance DXR efficacy. Potential underlying mechanisms were investigated independently, including increase in intracellular doxorubicin, temperature elevation, production of reactive oxygen species, changes in cell morphology, but none could explain the observed enhanced efficacy. Further work will be required to understand how non-inertial cavitation potentiates doxorubicin.

## Materials and Methods

### Reproducible generation of non-inertial cavitation

**Figure 10:**
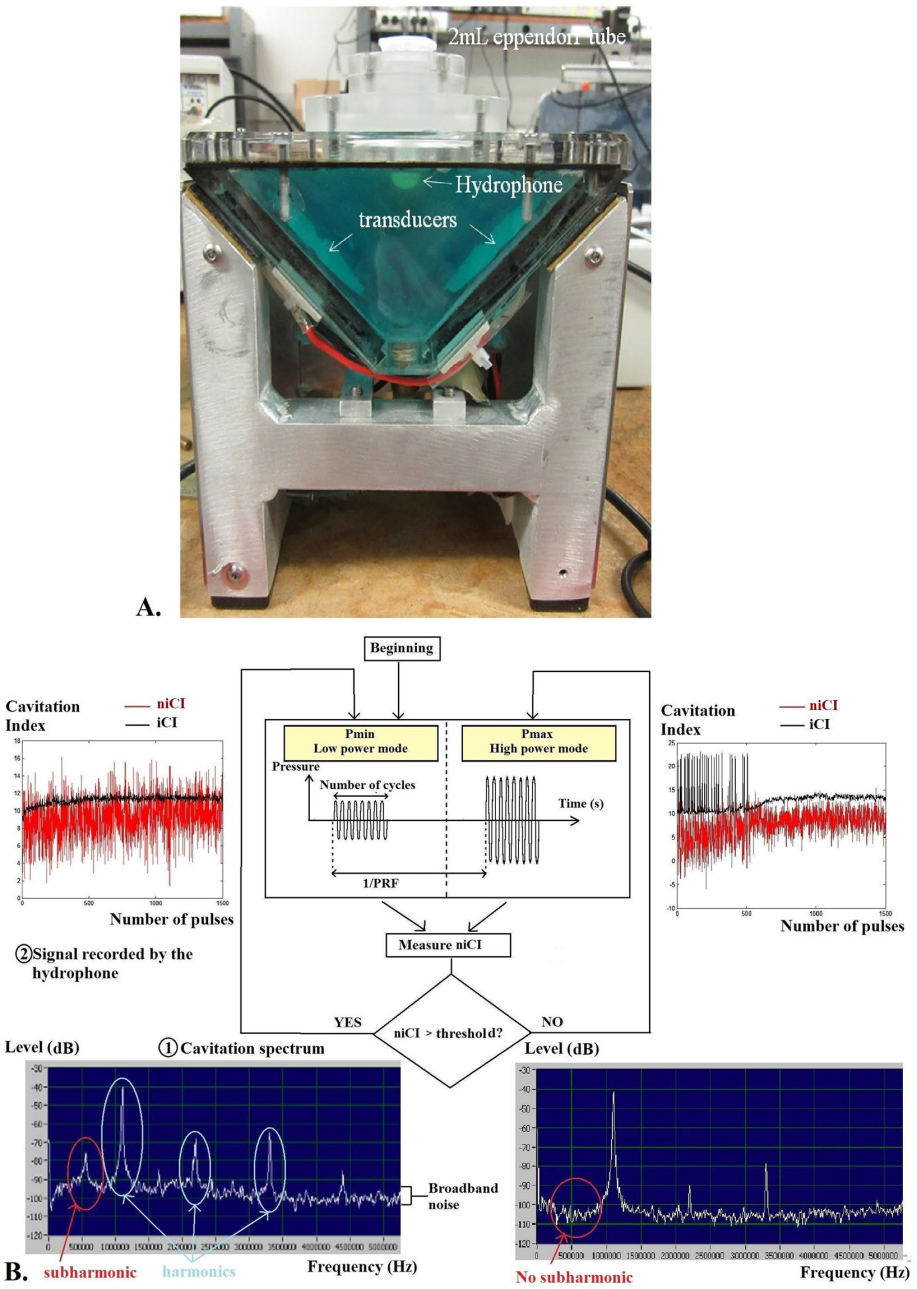
Cavitation setup. (**A**) The tank is filled with Ablasonic (**a**). The hydrophone (**c**) is placed in the solution between the two confocal transducers (**b**) and is directed towards the exposed sample contained in a 2 mL Eppendorf tube (**d**). (**B**) Schematic of the non-inertial cavitation control strategy. The control system is based on the alternation of high power pulses (Pmax) and low power pulses (Pmin). The level and type of cavitation are characterized by several indicators on the spectrum of the signal recorded by the hydrophone: occurrence of sub-harmonics (half the emission frequency) indicates non-inertial cavitation and high level of broadband noise indicates inertial cavitation. If the niCI (index of non-inertial cavitation) falls below a user-defined threshold (empirically defined at 3 in our case), a high power pulse is triggered to revive the cavitation cloud. The number of boosting pulses is recorded.

The cavitation device used in this study is composed of two confocal spherical PZ28 piezoceramic transducers (Ferroperm, Kvistgaard, Denmark) with curvature radius of 50 mm, separated by an angle of 90° and operated at 1.1 MHz (Fig. 10). This configuration favors the occurrence of cavitation^55^. The transducers were immersed in a tank filled with Ablasonic (EDAP-TMS, Vaulx-en-Velin, France), a cavitation-inhibitor liquid used to prevent acoustic cavitation outside the sonicated sample^56,57^.

For treatment, a 2 mL safe-lock micro test tube (Eppendorf, Hamburg, Germany), containing 650 µL of cell suspension, was positioned at the focal zone of the confocal device (Fig. 10A). The emission signal was generated with a National Instruments PXI-1031 device (National Instruments, Nanterre, France) driven by a LabVIEW software and powered by a 200 W amplifier (LA-200H EMV, Kalmus, Hong Kong, China). All ultrasound treatments were performed at room temperature.

Cavitation was monitored using an in-house hydrophone made with a polyvinylidene fluoride (PVDF) film (25 µm thickness, 10 mm in diameter) embedded in resin (AY103 Araldite+ 10% HY956) placed between the two focused transducers and directed towards the exposed sample. Signals were recorded at a sampling frequency of 33.3 MHz over segments of 16384 points.

Non-inertial cavitation activity can be maintained by reducing the acoustic pressure following creation of a bubble cavitation cloud by higher pressure^58^. In our experimental settings, preliminary experiments (internal data) have shown that driving the transducers in pulse mode with a pulse repetition frequency (PRF) of 25 Hz, and number of cycles of 6600, corresponding to a duty cycle (DC) = 15%, during an exposure time of 30 seconds, could generate non-inertial cavitation with a cell death limited to less than 30%. These settings were applied in the present study.

A non-inertial cavitation regulation system was implemented, using the recorded cavitation noise as input for a feedback loop amplitude control of the emitted signal. With this control system, two distinct modes can be emitted: a high-power mode or “bursting pulses” aimed at creating a population of bubbles, with a peak negative pressures (PNP) of 6.7 MPa (Pmax) at the focus, and a low-power mode or “regulation pulses” aimed at maintaining their oscillations, with a peak negative pressures (PNP) of 2.9 MPa (Pmin) at the focus.

The inertial and non-inertial cavitation contributions were separated based on the specific features in their frequency content for cavitation analysis (Fig. 10B). To quantify the level of inertial and non-inertial cavitation, two cavitation indexes (CI) were defined. The non-inertial cavitation index (niCI (dB)) value is monitored in real time and used to select the emission mode: if the niCI falls below the threshold associated with a lack of non-inertial cavitation, the system emits a bursting pulse to generate a new bubble cloud; if the niCI is above the threshold, the system emits low power mode to maintain bubbles oscillations. The niCI was calculated as the difference between the maximum power level in the 540–560 kHz sub-harmonic range and the mean base level obtained in the 560–600 kHz range. To calculate the inertial cavitation index, the average of the entire frequency spectrum in the 0.1–7.1 MHz range was first calculated in dB (re1mV) and then normalized by the base level, over the same frequency range, measured when the amplifier is turned on with no input signal^59^. The resulting cavitation index is therefore given in dB. The dB scale reduces the possible contribution of harmonic signal to the cavitation spectrum integration. Moreover, the length of the acquired signal is large enough to provide harmonics that are narrow enough in the frequency domain to be neglected. However, iCI is only slightly sensitive to harmonic peaks due to the presence of bubbles in medium and reflects the broadband noise due to inertial cavitation for iCI values greater than 6 dB^59–61^.

**Figure 11:**
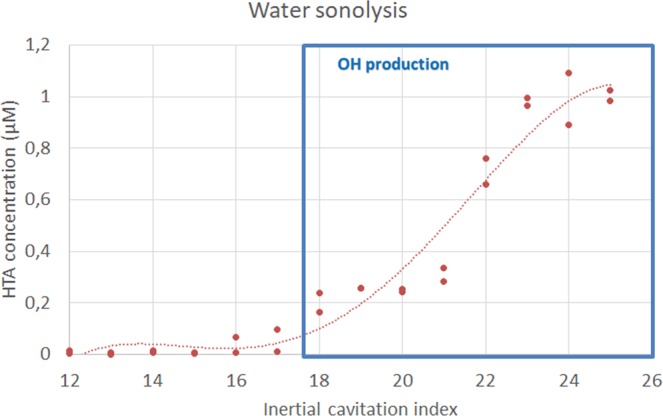
HTA concentration versus inertial cavitation indexes (iCI). The blue square represents values of iCI leading to HTA fluorescence (due to production of hydroxyl radicals reacting with terephtalate), a chemical dosimetry of inertial cavitation. Inertial cavitation occurs for iCI values higher than 18.

To validate the non-inertial cavitation regulation system, cavitation activity was also assessed by chemical dosimetry with terephthalate acid (Sigma Aldrich, Saint Quentin Fallavier, France). The terephthalate dosimeter is based on the formation of fluorescent hydroxyl-terephthalate (HTA) anions when the non-fluorescent terephthalate (TA) anions enter in contact with hydroxyl radicals (OH). Because inertial cavitation produces OH radicals, the dosimetry consists in exposing a TA solution to ultrasound-induced inertial cavitation, and measuring the HTA fluorescence being generated. HTA production, and therefore fluorescence intensity of the sonicated solution, will depend on sonication time and ultrasound intensity^62–64^ Test tubes containing 650 µL of TA at 20 mM were placed at the focus of the confocal device and sonicated for 30 seconds at different levels of iCI. After sonication, fluorescence of the samples was read using a spectrofluorometer (FluoroMax, Horiba Scientific, Kyoto, Japan), and HTA concentration in the samples was derived by normalization using the fluorescence level of a reference 2 µM HTA solution. As seen on Fig. 11, the cavitation threshold, above which a departure from baseline of HTA concentration can be attributable only to production of OH radicals, correspond to iCI typically above 18 dB. HTA concentration only depicts the sonochemical effect of inertial cavitation. However, for iCI below 18 dB, no rise of broadband noise (associated with inertial cavitation) or presence of higher subharmonics in the spectrum (associated with bubbles cloud oscillations) have been observed (Fig. 10).

Previous experiments have shown that inertial cavitation occurs for iCI typically higher than 18 dB (Fig. 11), and non-inertial cavitation for niCI typically higher than 3 dB, conservatives estimates to classify these acoustics emissions as significantly above noise levels.

### *In vitro* enhanced anti-tumor effect

#### Cells line and culture conditions

The 4T1 adherent murine mammary carcinoma cells, with a rapid doubling time of 23 h^65^, were cultured as a monolayer in T75 culture flasks in supplemented DMEM (10%FCS, 1% L-glutamine, 1% Penicillin-Streptomycine) and incubated at 37 °C in an humidified atmosphere (5% CO2, 95% O2). Passages were done every 3 days to prevent cells to exceed 90% confluence as overgrowth decreases their viability. All reagents were purchased from Invitrogen Life Technologies (Cergy-Pontoise, France). 4T1 cells were tested mycoplasma-free using Mycoalert^TM^ kit (Lonza, Cologne, Germany).

#### Chemicals

Doxorubicin hydrochloride 2 mg/mL (Accord Healthcare, Lille, France) was used at a concentration of 400 ng/mL. This dose, inducing 20% cell death at 72 h, was derived from estimation of the half maximal inhibitory concentration (IC50) from modelisation of the dose-response curve using MOSAIC macro^66^. For experiments involving hydroxyl radical scavengers, L-Histidine (Fluka, Sigma-Aldrich, Saint Louis MO, USA) at 20 mM and Mannitol (Fluka, Sigma-Aldrich, Saint Louis MO, USA) at 100 mM were used.

#### Combined treatment by doxorubicin and non-inertial cavitation

72 hours prior to the experiments, cells were plated at a density of 2. 10^6^ cells per T75 flask to reach 90% confluence on the days of the experiments. Cells were trypsinized with 0.25% trypsin/ 1 mM EDTA (Life Technologies, Cergy-Pontoise, France), centrifuged at 300 G during 5 min, washed once with 5 mL of PBS (Life Technologies, Cergy-Pontoise, France) to eliminate traces of Fetal Calf Serum (FCS) to avoid any association between DNA of dead cells and FCS under ultrasound exposure, and then resuspended in Opti-MEM Reduced-Serum Medium (Life Technologies, Cergy-Pontoise, France) at a concentration of 2 million cells per mL. The exact volume of Opti-MEM was determined after counting cells using an automated cell counter (Cellometer, Nexcelom Bioscience, Lawrence, MA).

The study design comprised four experimental conditions: (1) Non-Treated (NT) (2) DXR (DXR) (3) Ultrasound (US) (4) Combined ultrasound and DXR (US + DXR). For treatment, a volume of 650 µL of cells solution was pipetted in 2 mL Eppendorf tube using electronic pipettes. The tubes of DXR and US + DXR groups were supplemented with DXR at 400 ng/mL. Each tube of the US and US + DXR groups was positioned at the focus of the confocal system and sonication was applied using the parameters described earlier at room temperature. As differences in dissolved gas concentration in our culture medium (Opti-MEM) over the days may lead to differences in cavitation activity, we ensured the reproducibility of the non-inertial cavitation dose delivered. NT and DXR groups were manipulated the same way except for the ultrasound exposure. After treatment, a volume of 20 μL of cell suspension was collected for cell counting to assess immediate mortality. Then, for each sample, a volume containing 100 000 cells was collected. Those cells were plated in a 24-well plate, supplemented with 1 mL of culture medium, DXR at 400 ng/mL was added for the DXR and US + DXR groups and cells were incubated for 72 h at 37 °C.

To account for biological variability, this experiment was repeated six times, with 4 to 10 samples per group.

#### Cell viability

Cell viability was assessed by flow cytometry using a FACS LSR II (BD Bioscience, Franklin Lakes, NJ) and data processed using the FACS DIVA Software. Cells were stained with necrosis marker DAPI (DAPI, Sigma-Aldrich, Saint Louis MO, USA, intensity measured with the 405 nm blue laser), and apoptosis marker Annexin V (Annexin V APC, Annexin V apoptosis detection kit APC, eBiosciences, Paris, France, intensity measured with the 633 nm red laser), and assessed viable if negative for both markers. Flow cytometry was performed 1 h and 72 h after sonication to assess short-term cytotoxicity due to ultrasound treatment alone and combined treatment cytotoxicity, respectively. The 1 h time point assessment was performed on cells kept in suspension after the treatment: 300 µL of the 650 µL of cell suspension contained in the Eppendorf tubes were collected and directly placed in FACS tubes. For the 72 h time point, the supernatant was removed from culture wells, wells were washed with 500 µL PBS, cells were trypsinized and collected, centrifuged (300 G for 5 min), washed with 1 mL PBS, centrifuged again, washed with 500 µL of Annexin V APC Buffer, centrifuged, incubated with Annexin V APC (Dilution 1/100, volume: 100 μL/sample) in the dark for 10 minutes, and finally incubated with DAPI (Dilution 1/500, concentration: 2 μg/mL, volume: 200 μL/sample) and resuspended in 300 µL of PBS, before being analyzed by FACS.

#### Cell counting

Cell counting was performed 1 h and 72 h after sonication using a Cellometer (Nexcelom Bioscience, Lawrence, MA). Automated cell counting was always checked manually to prevent potential device inaccuracies that could be caused by cellular aggregates. Special attention was placed on samples treated with DXR or US + DXR at 72 h, as the cell concentration in these samples were on the lower end of the device’s sensitivity. To ensure that the immediate mortality was inferior to the limit value of 30%, the dead cells percentage was measured immediately after sonication. After 72 h of culture, a cell growth index was calculated as the absolute number of cells divided by the initial number of cells reseeded after treatment (100 000 cells). An index less than 1 indicates inhibition of cell proliferation and/or cell death, while an index greater than 1 indicates continuous cell proliferation.

### Mechanistic study

A set of experiments was designed to investigate the role of cavitation, DXR internalization (sonoporation), temperature increase, generation of free radicals, and changes in cell morphology, in the cell viability and proliferation changes after the combined treatment.

#### Influence of cavitation

To investigate whether cavitation is required to induce the observed biological response, an experiment was designed to deliver the same acoustic energy, with or without cavitation. In the first setting, ultrasound treatment parameters were as previously described. In the second setting, the bursting pulses were omitted to prevent onset of cavitation and cells treated only with the regulation pulses. Because the bursting pulses are responsible for a very small fraction of the total delivered acoustic energy to the cells (see results section), it was considered that cells were treated with equivalent acoustic energy dose in both settings. The presence or absence of cavitation was monitored in both settings via quantification of the sub-harmonics component in the spectrum of the hydrophone-recorded signals. The experimental design was made of three conditions with 5 samples for each: (1) DXR only (DXR), (2) DXR + US without cavitation (DXR + US-NoCav), (3) DXR + US with cavitation (DXR + US-Cav). Cell viability was assessed 1 h and 72 h post-treatment as previously described (subsection *Cell viability*).

#### Analysis of DXR internalization

Doxorubicin is fluorescent (Excitation: 470 nm, Emission: 590 nm^67^), and its internalization was assessed by measuring the mean fluorescence intensity of cells using flow cytometry (intensity measured with the 488 nm blue laser). The fluorescence intensity of internalized DXR was assessed 1 h and 72 h after sonication.

To account for biological variability, this experiment was repeated five times, with 4 to 10 samples per group.

#### Influence of temperature increase

To measure temperature changes induced by the US treatment, a thermocouple was placed in the Eppendorf tube during sonication. To avoid perturbations in temperature measurements due to cavitation and to artifacts from direct sonication on the thermocouple, the thermocouple was placed a few mm away from the focal zone, assuming the temperature in the solution to be homogeneous due to the strong acoustic streaming. The temperature increased of 2 °C during the 30 s sonication, rising from 24 °C (room temperature (RT)) to 26 °C.

**Figure 12:**
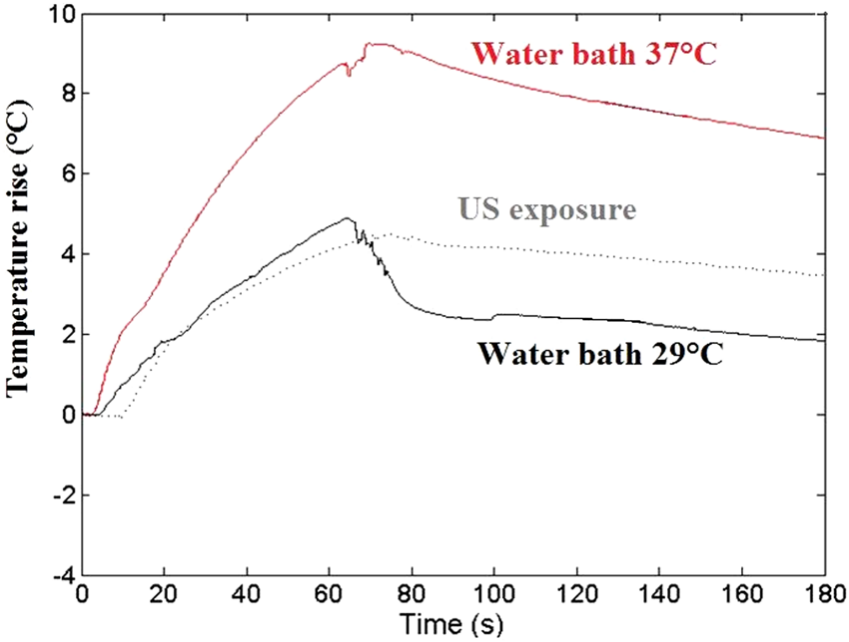
Temperature curves. Temperature curves measured by thermocouples in the Eppendorf tubes submitted to 30 s US exposure or placed in a water bath (T = 29 °C or T = 37 °C).

To study specifically the potential impact of this temperature increase on cell viability, tubes containing cells solution were placed for 30 s in water bath heated either at 29 °C, (that induced 2 °C temperature increase over the 30 s course), or at 37 °C to simulate physiological conditions (Fig. 12). Tubes were removed from the water bath after a time interval equivalent to US exposure time and allowed to cool at RT. Three experimental conditions were studied, with 4 samples per condition: (1) DXR at RT of 24 °C (DXR-RT), (2) DXR heated at 29 °C (DXR-29), (3) DXR heated at 37 °C (DXR-37). Cell viability was assessed 72 h post-heating as previously described (subsection *Cell viability*).

#### Effect of reactive oxygen species (ROS)

The production of ROS was assessed with flow cytometry by using H2DCFDA (Fischer Scientific, Illkirch, France), a fluorescent marker that becomes fluorescent when linked to ROS^68^. Because the emission wavelength of H2DCFDA is closed to that of DXR (intensity measured in the 488 nm blue laser), compensation was performed prior to FACS analysis. The experimental design was made of four conditions of 6 samples each: (1) Non-treated (NT) (2) DXR (DXR) (3) US-treated (US) (4) Combination of US and DXR (US + DXR). Cell viability was assessed similarly as previously described. 1 mL of H2DCFDA diluted at 1:1000 (volume: 50 μL/sample) was added between adding the DAPI and Annexin markers and washing the cells with PBS. Cells were then incubated for 30 minutes at 37 °C, washed twice with 1 mL PBS, incubated with 1 mL of DMEM at 37 °C for 10 minutes (step of fixation of the reactive) and washed twice with 1 mL PBS.

In ROS experiments, cells were treated in the presence of a ROS scavenger, L-Histidine, to specifically mitigate the effects of hydroxyl^69^. Before treatment, scavenger L-Histidine at 20 mmol/L was added to the cell suspension. The experimental design was made of 8 groups, with 8 samples per group: (1) Non-Treated (NT), (2) DXR (DXR), (3) L-Histidine treated (Hist), (4) Combination of DXR and L-Histidine (DXR + Hist), (5) US-treated (US), (6) Combination of DXR and US (US + DXR), (7) Combination of US and L-Histidine (US + Hist), (8) Combination of DXR, US and L-Histidine (US + DXR + Hist).

#### Cell microstructure

Modifications in cell microstructure were observed by transmission electron microscopy (Jeol 1400 JEM, Tokyo, Japan). Ultrasound cavitation and activation of apoptotic pathways are expected to induce membrane pore complexes, nuclear pore complexes and DNA damages as a result of compaction and marginalization of nuclear chromatin, convolution of cytoplasmic and nuclear membranes and condensation of cytoplasm^32^. The experimental design had four arms of 4 samples: (1) Non-Treated (NT), (2) DXR-Treated (DXR), (3) US-Treated (US), (4) Combination of DXR and US (US + DXR). After treatment, cells were fixed in glutaraldehyde 2%. The samples were washed three times in saccharose 0.4 M and Na C-HCl Cacodylate 0.2 M pH 7.4 for 1 hour at 4 °C, and postfixed with 2% OsO4 and Na C-HCl Cacodylate 0.3 M pH 7.4 30 minutes at room temperature. Cells were then dehydrated with an increasing ethanol gradient (5 minutes in 30%, 50%, 70%, 95% and 3 times for 10 minutes in absolute ethanol). Impregnation was performed with Epon A (50%) plus Epon B (50%) plus DMP30 (1.7%). Inclusion was obtained by polymerization at 60 °C for 72 hours. Ultrathin sections (approximately 70 nm thick) were cut on a ultracut UC7 (Leica) ultramicrotome, mounted on 200 mesh copper grids coated with 1:1000 polylysine, and stabilized for one day at room temperature and finally, contrasted with uranyl acetate. Sections were examined with a Jeol 1400 JEM (Tokyo, Japan) transmission electron microscope equipped with an Orius 600 camera and Digital Micrograph (magnifications x10, x15, x30). Nuclear pore complexes (NPC) were identified as nuclear membrane thinning and counted for each experimental group.

### Statistical analysis

Data are presented as means +/− standard deviation (SD). Statistical analyses were performed using the R Software (R Core Team (2015)). Non-parametric Kruskal-Wallis’s test with Dunn post hoc test was used. A p-value less than 0.05 was deemed statistically significant. The stars are only intended to flag levels of significance for three of the most commonly used levels. If a p-value is less than 0.05, 0.01 or 0.001, it is flagged with respectively one star (*), two stars (**) or three stars (***).

To correct for disparity in viability between the different experiments, which can be caused by different experimental factors such as transportation of the cells from US treatment facility to FACS facility or the different passages of the cell lines, data were centered so that the average viability of the control groups for individual experiments was equal to the overall viability of all the measured control groups, an overall measure of viability for this cell line. This normalization does not modify data distribution.

## Data Availability

The datasets generated during and/or analysed during the current study are available in the Open Science Framework repository. [https://osf.io/w4tzn/?view_only=cb74af5da54a4089b060c8310f953a5f].

## References

[1] Wang S (2004). Doxorubicin induces apoptosis in normal and tumor cells via distinctly different mechanisms. intermediacy of H(2)O(2)- and p53-dependent pathways. J. Biol. Chem..

[2] Bakkali H, Marchal C, Lesur-Schwander A, Verhaeghe J-L (2003). Breast cancer in women thirty years old or less. Cancer Radiother. J. Soc. Francaise Radiother. Oncol..

[3] Carvalho C (2009). Doxorubicin: the good, the bad and the ugly effect. Curr. Med. Chem..

[4] Swift LP, Rephaeli A, Nudelman A, Phillips DR, Cutts SM (2006). Doxorubicin-DNA adducts induce a non-topoisomerase II-mediated form of cell death. Cancer Res..

[5] Dobson J, Dobson J (2006). Magnetic nanoparticles for drug delivery. Drug Develop. Res. 67, 55–60. Drug Dev. Res..

[6] Trendowski M (2014). The promise of sonodynamic therapy. Cancer Metastasis Rev.

[7] Saad AH, Hahn GM (1989). Ultrasound enhanced drug toxicity on Chinese hamster ovary cells *in vitro*. Cancer Res..

[8] Loverock P, ter Haar G, Ormerod MG, Imrie PR (1990). The effect of ultrasound on the cytotoxicity of adriamycin. Br. J. Radiol..

[9] Yu T, Wang Z, Jiang S (2001). Potentiation of cytotoxicity of adriamycin on human ovarian carcinoma cell line 3AO by low-level ultrasound. Ultrasonics.

[10] Stride EP, Coussios CC (2010). Cavitation and contrast: the use of bubbles in ultrasound imaging and therapy. Proc. Inst. Mech. Eng. [H].

[11] Ferrara K, Pollard R, Borden M (2007). Ultrasound microbubble contrast agents: fundamentals and application to gene and drug delivery. Annu. Rev. Biomed. Eng..

[12] Sennoga CA (2017). Microbubble-mediated ultrasound drug-delivery and therapeutic monitoring. Expert Opin. Drug Deliv..

[13] Carpentier A (2016). Clinical trial of blood-brain barrier disruption by pulsed ultrasound. Sci. Transl. Med..

[14] Mainprize T (2019). Blood-Brain Barrier Opening in Primary Brain Tumors with Non-invasive MR-Guided Focused Ultrasound: A Clinical Safety and Feasibility Study. Sci. Rep.

[15] O’Reilly MA, Hynynen K (2012). Blood-brain barrier: real-time feedback-controlled focused ultrasound disruption by using an acoustic emissions-based controller. Radiology.

[16] Sun T (2017). Closed-loop control of targeted ultrasound drug delivery across the blood–brain/tumor barriers in a rat glioma model. Proc. Natl. Acad. Sci..

[17] Alheshibri M, Qian J, Jehannin M, Craig VSJ (2016). A History of Nanobubbles. Langmuir.

[18] Lafond, M., Watanabe, A., Yoshizawa, S., Umemura, S. & Tachibana, K. Cavitation-threshold Determination and Rheological-parameters Estimation of Albumin-stabilized Nanobubbles. *Sci*. *Rep*. **8** (2018). 10.1038/s41598-018-25913-8 PMC5945894 29748624

[19] Pellow C, Goertz DE, Zheng G (2018). Breaking free from vascular confinement: status and prospects for submicron ultrasound contrast agents. Wiley Interdiscip. Rev. Nanomed. Nanobiotechnol..

[20] Kwan JJ (2015). Ultrasound-Propelled Nanocups for Drug Delivery. Small Weinh. Bergstr. Ger..

[21] Cavalli R (2015). Preparation and *in vitro* characterization of chitosan nanobubbles as theranostic agents. Colloids Surf. B Biointerfaces.

[22] Hallow DM, Mahajan AD, McCutchen TE, Prausnitz MR (2006). Measurement and correlation of acoustic cavitation with cellular bioeffects. Ultrasound Med. Biol..

[23] Feng Y, Tian Z, Wan M (2010). Bioeffects of low-intensity ultrasound *in vitro*: apoptosis, protein profile alteration, and potential molecular mechanism. J. Ultrasound Med. Off. J. Am. Inst. Ultrasound Med..

[24] Bouakaz A, Zeghimi A, Doinikov AA (2016). Sonoporation: Concept and Mechanisms. Adv. Exp. Med. Biol..

[25] Meijering BDM (2009). Ultrasound and microbubble-targeted delivery of macromolecules is regulated by induction of endocytosis and pore formation. Circ. Res..

[26] Pitt WG, Husseini GA, Staples BJ (2004). Ultrasonic drug delivery–a general review. Expert Opin. Drug Deliv..

[27] Escoffre J-M, Zeghimi A, Novell A, Bouakaz A (2013). *In-vivo* gene delivery by sonoporation: recent progress and prospects. Curr. Gene Ther..

[28] Furusawa Y (2014). Effects of therapeutic ultrasound on the nucleus and genomic DNA. Ultrason. Sonochem.

[29] Chen X, Wan JMF, Yu ACH (2013). Sonoporation as a cellular stress: induction of morphological repression and developmental delays. Ultrasound Med. Biol..

[30] Kondo T, Yoshii G (1985). Effect of intensity of 1.2 MHz ultrasound on change in DNA synthesis of irradiated mouse L cells. Ultrasound Med. Biol..

[31] Sakiyama Y, Panatala R, Lim RYH (2017). Structural dynamics of the nuclear pore complex. Semin. Cell Dev. Biol..

[32] Al-Bahlani S, Al-Dhahli B, Al-Adawi K, Al-Nabhani A, Al-Kindi M (2017). Platinum-Based Drugs Differentially Affect the Ultrastructure of Breast Cancer Cell Types. BioMed Res. Int.

[33] Chen C, Liu Y, Maruvada S, Myers M, Khismatullin D (2012). Effect of ethanol injection on cavitation and heating of tissues exposed to high-intensity focused ultrasound. Phys. Med. Biol..

[34] Doroshow JH (1983). Anthracycline antibiotic-stimulated superoxide, hydrogen peroxide, and hydroxyl radical production by NADH dehydrogenase. Cancer Res..

[35] Mizutani H, Tada-Oikawa S, Hiraku Y, Kojima M, Kawanishi S (2005). Mechanism of apoptosis induced by doxorubicin through the generation of hydrogen peroxide. Life Sci..

[36] Desjouy C (2015). Counterbalancing the use of ultrasound contrast agents by a cavitation-regulated system. Ultrason. Sonochem.

[37] Mashiko D (2018). Estimation of sonodynamic treatment region with sonochemiluminescence in gel phantom. Jpn. J. Appl. Phys..

[38] Al-Ghamdi SS (2008). Time and dose dependent study of doxorubicin induced DU-145 cytotoxicity. Drug Metab. Lett.

[39] Schlicher RK (2006). Mechanism of intracellular delivery by acoustic cavitation. Ultrasound Med. Biol..

[40] Lammertink B, Deckers R, Storm G, Moonen C, Bos C (2015). Duration of ultrasound-mediated enhanced plasma membrane permeability. Int. J. Pharm..

[41] Yudina A, Lepetit-Coiffé M, Moonen CTW (2011). Evaluation of the temporal window for drug delivery following ultrasound-mediated membrane permeability enhancement. Mol. Imaging Biol. MIB Off. Publ. Acad. Mol. Imaging.

[42] Dalmark M, Storm HH (1981). A Fickian diffusion transport process with features of transport catalysis. Doxorubicin transport in human red blood cells. J. Gen. Physiol..

[43] Janssen F-PEM, Bouten CVC, van Leeuwen GMJ, van Steenhoven AA (2008). Effects of temperature and doxorubicin exposure on keratinocyte damage *in vitro*. In Vitro Cell. Dev. Biol. Anim..

[44] Lane P, Vichi P, Bain DL, Tritton TR (1987). Temperature Dependence Studies of Adriamycin Uptake and Cytotoxicity. Cancer Res..

[45] Lyon PC (2018). Safety and feasibility of ultrasound-triggered targeted drug delivery of doxorubicin from thermosensitive liposomes in liver tumours (TARDOX): a single-centre, open-label, phase 1 trial. Lancet Oncol..

[46] Rosenthal I, Sostaric JZ, Riesz P (2004). Sonodynamic therapy–a review of the synergistic effects of drugs and ultrasound. Ultrason. Sonochem.

[47] Umemura S (1997). Sonodynamically-induced *in vitro* cell damage enhanced by adriamycin. Cancer Lett..

[48] Meredith A-M, Dass CR (2016). Increasing role of the cancer chemotherapeutic doxorubicin in cellular metabolism. J. Pharm. Pharmacol..

[49] Vaškovicová N, Druckmüllerová Z, Janisch R, Škorpíková J, Mornstein V (2013). Effects of therapeutic ultrasound on the nuclear envelope and nuclear pore complexes. J. Appl. Biomed..

[50] Zhong W, Sit WH, Wan JMF, Yu ACH (2011). Sonoporation induces apoptosis and cell cycle arrest in human promyelocytic leukemia cells. Ultrasound Med. Biol..

[51] Zhong W (2013). Induction of endoplasmic reticulum stress by sonoporation: linkage to mitochondria-mediated apoptosis initiation. Ultrasound Med. Biol..

[52] Hundt W, Yuh EL, Bednarski MD, Guccione S (2007). Gene Expression Profiles, Histologic Analysis, and Imaging of Squamous Cell Carcinoma Model Treated with Focused Ultrasound Beams. Am. J. Roentgenol.

[53] Ashley N, Poulton J (2009). Mitochondrial DNA is a direct target of anti-cancer anthracycline drugs. Biochem. Biophys. Res. Commun..

[54] Eom Y-W (2005). Two distinct modes of cell death induced by doxorubicin: apoptosis and cell death through mitotic catastrophe accompanied by senescence-like phenotype. Oncogene.

[55] Lafond M, Prieur F, Chavrier F, Mestas J-L, Lafon C (2017). Numerical study of a confocal ultrasonic setup for cavitation creation. J. Acoust. Soc. Am..

[56] Chettab K (2015). Spatial and Temporal Control of Cavitation Allows High *In Vitro* Transfection Efficiency in the Absence of Transfection Reagents or Contrast Agents. PLoS One.

[57] Chettab K (2017). Doxorubicin Delivery into Tumor Cells by Stable Cavitation without Contrast Agents. Mol. Pharm..

[58] Mestas J-L, Lenz P, Cathignol D (2003). Long-lasting stable cavitation. J. Acoust. Soc. Am..

[59] Frohly J, Labouret S, Bruneel C, Looten-Baquet I, Torguet R (2000). Ultrasonic cavitation monitoring by acoustic noise power measurement. J. Acoust. Soc. Am..

[60] Sabraoui A, Inserra C, Gilles B, Béra J-C, Mestas J-L (2011). Feedback loop process to control acoustic cavitation. Ultrason. Sonochem.

[61] Reslan L, Mestas J-L, Herveau S, Béra J-C, Dumontet C (2010). Transfection of cells in suspension by ultrasound cavitation. J. Control. Release Off. J. Control. Release Soc.

[62] Villeneuve L, Alberti L, Steghens J-P, Lancelin J-M, Mestas J-L (2009). Assay of hydroxyl radicals generated by focused ultrasound. Ultrason. Sonochem.

[63] Price GJ, Duck FA, Digby M, Holland W, Berryman T (1997). Measurement of radical production as a result of cavitation in medical ultrasound fields. Ultrason. Sonochem.

[64] McLean JR, Mortimer AJ (1988). A cavitation and free radical dosimeter for ultrasound. Ultrasound Med. Biol..

[65] Pulaski BA, Ostrand‐Rosenberg S (2001). Mouse 4T1 Breast Tumor Model. Curr. Protoc. Immunol..

[66] Charles S, Veber P, Delignette-Muller ML (2018). MOSAIC: a web-interface for statistical analyses in ecotoxicology. Environ. Sci. Pollut. Res..

[67] Laginha KM (2005). Determination of Doxorubicin Levels in Whole Tumor and Tumor Nuclei in Murine Breast Cancer Tumors. Clin. Cancer Res..

[68] Ameziane El Hassani, R. & Dupuy, C. Detection of Intracellular Reactive Oxygen Species (CM-H2DCFDA). *BIO-Protoc*. **3** (2013).

[69] Yu T, Bai J, Hu K, Wang Z (2003). The effect of free radical scavenger and antioxidant on the increase in intracellular adriamycin accumulation induced by ultrasound. Ultrason. Sonochem..

